# A Δ42PD1 fusion-expressing DNA vaccine elicits enhanced adaptive immune response to HIV-1: the key role of TLR4

**DOI:** 10.1186/s12985-022-01909-9

**Published:** 2022-11-01

**Authors:** Lin Cheng, Xian Tang, Yun He, Bin Ju, Hui Wang

**Affiliations:** 1 Institute for Hepatology, National Clinical Research Center for Infectious Disease, Shenzhen Third People’s Hospital, Shenzhen, 518112 Guangdong Province China; 2 Department of Infectious Diseases, Shenzhen Third People’s Hospital, Shenzhen, 518112 Guangdong Province China; 3 The Second Affiliated Hospital, School of Medicine, Southern University of Science and Technology, Shenzhen, 518112 Guangdong Province China

**Keywords:** DNA vaccine, Δ42PD1, TLR4, Dendritic cell, Immunogenicity

## Abstract

Since its discovery in the 1990s, the DNA vaccine has been of great interest because of its ability to elicit both humoral and cellular immune responses while showing relative advantages regarding producibility, stability and storage. However, when applied to human subjects, inadequate immunogenicity remains as the greatest challenge for the practical use of DNA vaccines. In this study, we generated a DNA vaccine Δ42PD1-P24 encoding a fusion protein comprised of the HIV-1 Gag p24 antigen and the extracellular domain of murine Δ42PD1, a novel endogenous Toll-like receptor 4 (TLR4) agonist. Using a mouse model, we found that Δ42PD1-P24 DNA vaccine elicited a higher antibody response and an increased number of IFN-γ-producing CD4 and CD8 T cells. Moreover, mice with Δ42PD1-P24 DNA vaccination were protected from a subcutaneous challenge with murine mesothelioma cells expressing the HIV-1 p24 antigen. Importantly, the Δ42PD1-mediated enhancement of immune responses was not observed in TLR4 knockout mice. Collectively, these data demonstrate that the immunogenicity and efficacy of DNA vaccines could be improved by the fusion of the extracellular domain of Δ42PD1 to target the immunogen to dendritic cells.

**Supplementary Information:**

The online version contains supplementary material available at 10.1186/s12985-022-01909-9.

## Introduction

Compared with traditional vaccines, including inactivated, subunit or recombinant protein vaccines, DNA vaccines have significant advantages in eliciting both humoral and cellular immune responses [1] which is essential for viral elimination [2]. Accordingly, DNA vaccines have been extensively investigated to fight against challenging infectious diseases, particularly human immunodeficiency virus type 1 (HIV-1) and emerging pathogens. The HIV-1 DNA vaccine was one of the first DNA vaccines tested in non-human primates [1, 3] and the first tested in humans [4]. DNA vaccines for Middle East Respiratory Syndrome (MERS) and severe acute respiratory syndrome (SARS) were well-tolerated and induced neutralizing antibodies and T cell immune responses in clinical trials [5, 6]. The rapid spread of severe acute respiratory syndrome coronavirus 2 (SARS-CoV-2) highlights the urgent development of effective vaccines. Multiple DNA vaccines against COVID-19 have been tested in human [7], and the first DNA vaccine for human use has been approved by Indian authorities to address the urgent need for a medical countermeasure to prevent the further dissemination of SARS-CoV-2 [8].

DNA vaccines mainly transfect muscle cells which are not able to present antigen through MHC class II as needed to induce CD4 helper T cells, resulting in poor antigen presentation and sub-optimal immunogenicity. One promising strategy to overcome this obstacle is the fusion expression of a dendritic cell (DC)-targeting molecule in the DNA vaccine construct. Many such targeting approaches have been successful in mouse models, utilizing a wide range of targeting molecules including PD-L1/2, Cle9A, Flt3 and DEC205 [9].

Δ42PD1, a novel alternatively spliced PD-1 (programmed cell death protein-1) isoform, does not engage PD-L1/2 but instead interacts with Toll-like receptor 4 (TLR4), triggering dendritic cells to produce proinflammatory cytokines [10–12]. We, therefore, assume that a DNA vaccine encoding Δ42PD1 fused with the immunogen of interest can enhance the immune responses by targeting the antigen directly to dendritic cells via engaging TLR4. In the present study, we tested this assumption by incorporating the extracellular domain of Δ42PD1 into the HIV-1 P24 DNA vaccine. We found that this approach greatly enhances antigen immunogenicity and protective efficacy in vivo.

## Materials and methods

### Plasmid and vaccines

The carrier plasmid pVAX1 for constructing the DNA vaccines was provided by Prof. Zhiwei Chen from The University of Hong Kong. For vaccine P24, the coding sequence of HIV-1 Gag p24 (GenBank Accession No. DQ007902) was codon optimized, synthesized with the human tissue plasminogen activator (tPA) (GenBank Accession No. NM_000930.3) signal peptide (M1-P22) at the N terminus and two restriction endonuclease cleavage sites HindIII/XhoI outside the open reading frame. For vaccine Δ42PD1-P24, the extracellular domain of mouse Δ42PD1 (S20-V156) was added between the tPA signal peptide and the p24 antigen based on vaccine P24. Both synthesized sequences were cloned into T-vector (TaKaRa) by Sangon Biotech. To construct DNA vaccines, the open reading frames were then cut off from T-vectors, and cloned into pVAX1 between HindIII/XhoI using T4 ligase. pVAX1 vectors and the constructed vaccines were prepared using EndoFree Plasmid Giga Kit (Qiagen).

### Mouse immunization and tumor challenge

Six to eight weeks old female BALB/c mice (Guangdong Medical Laboratory Animal Center) and TLR4 knockout C57BL/6 mice generated using CRISPR/Cas9 technique (Cyagen Biosciences) were bred under standard pathogen-free conditions. For DNA vaccination, each mouse was immunized with 20 μg of endotoxin-free DNA vaccines or pVAX1 vector twice at four-week intervals intramuscularly plus electroporation as we described previously [13]. Two weeks after the last immunization, mice were euthanized for immunogenicity analysis. For tumor challenge, two weeks after the 2nd immunization, 5 × 10^5^ AB1-Gag cells were inoculated subcutaneously in the right flank of mice. The tumor size was measured each two-four days. Tumor size based on caliper measurement was calculated by the modified ellipsoidal formula, tumor size = (length × width^2^)/2. Mice were sacrificed at the endpoint (21 days post-challenge). The mice were raised under specific pathogen-free (SPF) conditions and were fed a normal diet.

### Enzyme linked immunosorbent assay

For HIV-1 Gag p24 determination, two days after transfection, p24 antigen in cell culture media was detected using HIV Type 1 p24 Antigen ELISA 2.0 (ZeptoMetrix) following kit instructions.

For plasma antibody determination, recombinant HIV-1 p24 protein (Abcam, 0.5 μg/ml) was coated in 96-well EIA/RIA plates overnight at 4 °C. The plates were blocked with 5% skim milk at 37 °C for 1 h. After washing, plasmas (two-fold serially diluted from 1:50 or 50-fold diluted) were added and incubated for 1 h at 37 oC. After washing, goat anti-mouse IgG H&L (HRP) secondary antibody (Abcam) diluted 1:50,000 was added. Plates were then incubated at 37 ^o^C for 1 h. After extensive washes, 100 µl of the substrate was added and incubated for 10 min at room temperature, followed by adding 100 µl of stop solution. The optical density at 450 nm was determined using a Varioskan LUX (Thermo Scientific).

### Interferon gamma (IFN-γ) ELISpot assay

Splenocytes from immunized mice were isolated two weeks after the last immunization using 70 μm cell strainers (BD Sciences) and Mouse Lymphocyte Separation Medium (Dakewe Biotech). P24 peptide pool containing 55 peptides of 15 amino acid residues in length overlapping by 11 residues, the BALB/c H2-K^d^ CD4 T cell epitope (TNNPPIPVGEIYKRWIILGL) and the CD8 T cell epitope (AMQMLKETI) were synthesized by Genscript Biotech. ELISpot assay was performed as we previously described [14].

### Flow cytometry

For tetramer staining, lithium-heparin anticoagulated whole blood samples were stained with anti-mouse CD3, anti-mouse CD8 antibodies (BioLegend) and MHC-I H-2K^d^ HIV Gag Tetramer-AMQMLKETI-APC (MBL) for 30 min at 4 °C. Red blood cells were depleted by cell lysis buffer (BD Biosciences). Cells were then washed twice with PBS containing 2% FBS, and re-suspended in 1% paraformaldehyde. Samples were acquired using a BD FACSCanto II cytometer (BD Biosciences) and the data were analyzed with FlowJo software (Tree Star).

### Statistical analysis

The GraphPad Prism 8.0 software was used for statistical analysis, and *P*-value less than 0.05 was considered significant. The data were all expressed as the mean and the standard error of the mean (Mean ± SEM).

## Results

**Fig. 1:**
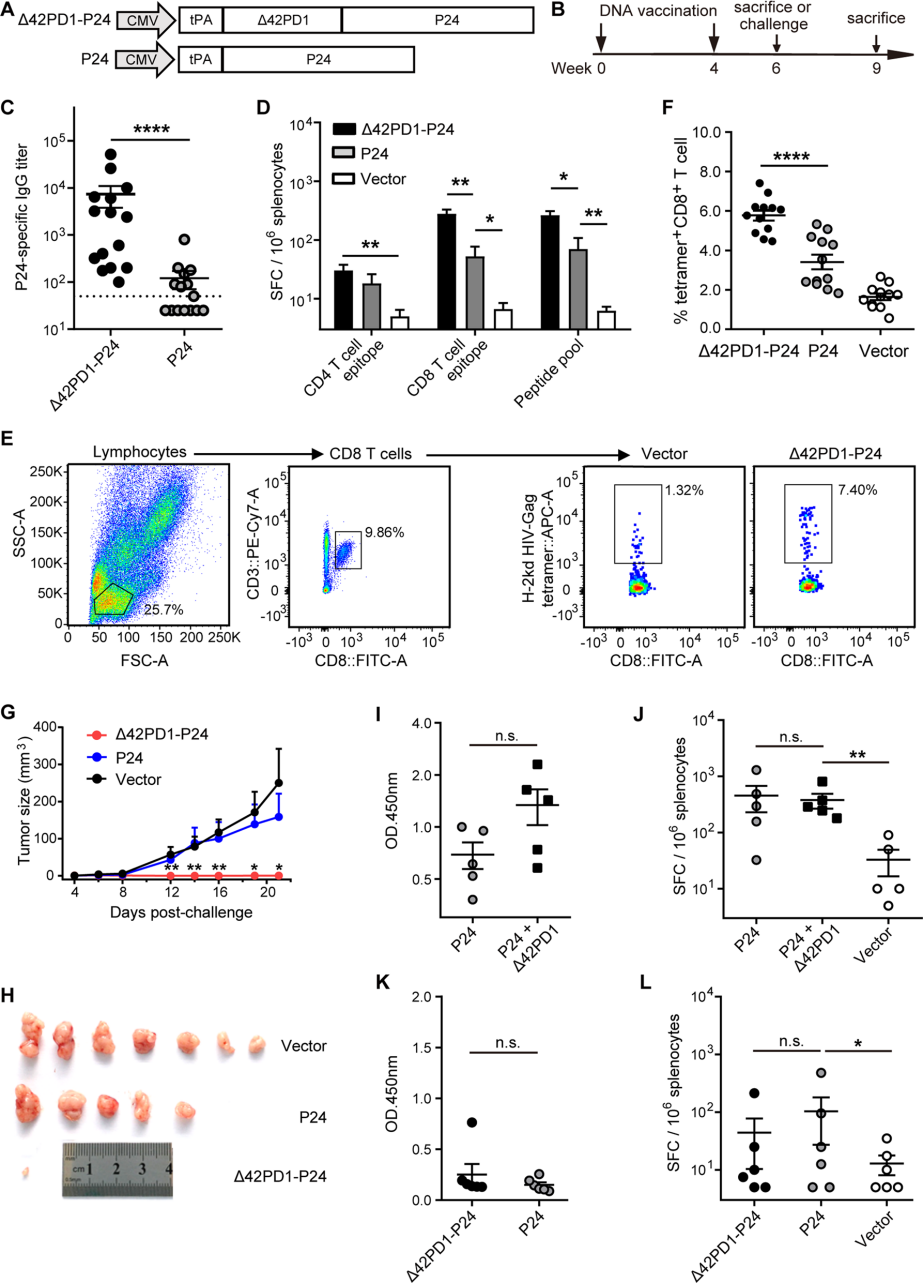
Δ42PD1 enhanced adaptive immune response against fusion expressed p24 antigen. **A** Schematic representation of DNA vaccine constructs. **B** Schematic representation of mice immunization schedule. **C** ELISA analysis of anti-p24 plasma antibody titers post-immunization of indicated vaccines (n = 15). The dotted line indicates the lower detection limit. **D** ELISpot analysis of p24-specific CD4^+^ and CD8^+^ T cell responses by stimulating splenocytes of immunized mice with indicated p24 peptides (n = 6). SFC, spot forming cells. **E** The gating strategy for tetramer^+^ CD8 T cells and representative plots. **F** The frequency of tetramer^+^ CD8 T cells in indicated vaccination groups (n = 15). **G** Tumor size in immunized mice was estimated by two-dimensional caliper measurement post-injection of AB1-Gag cells (n = 8). **H** Tumor-bearing mice were sacrificed at day 21 post-challenge, and the tumors were shown. **I**, **J** Mice were vaccinated with the mixture of the P24 vaccine with vector (P24) or pVAX-Δ42PD1 (P24 + Δ42PD1), or with the vector alone (n = 5). Two weeks after the last immunization, anti-p24 plasma antibody level **I** and antigen-specific T cells response to stimuli of p24 peptide pool **J** were determined. **K**, **L** TLR4 knockout mice were vaccinated with P24, Δ42PD1-P24 vaccines or pVAX1 vector (n = 6). Two weeks after the last immunization, anti-p24 plasma antibody level **K** and antigen-specific T cells response to stimuli of p24 peptide pool **L** were determined. **C**, **D**, **F**, **G**, **I**, **L** Data was represented as mean ± SEM of samples in the group. All statistical analyses were performed by student's *t*-test (Mann-Whitney test) using GraphPad Prism 8.0 software. *P < 0.05, **P < 0.01, ****P < 0.0001, n.s., not significant

Two DNA vaccines, named P24 and Δ42PD1-P24, were generated by synthesizing codon-optimized fusion open reading frames (ORFs) of the human tissue plasminogen activator (tPA) signal peptide, HIV-1 Gag p24 with or without the extracellular domain of mouse Δ42PD1. Then the fusion ORFs were inserted into the pVAX1 vector under the cytomegalovirus (CMV) promoter (Fig. 1A). The expression of the ORFs was confirmed by RT-PCR and ELISA after transfection of HEK-293 T cells with the constructs (Additional file 1: Fig. S1).

To explore the effect of Δ42PD1 fusion on eliciting p24-specific immune responses, BALB/c mice were immunized with P24, Δ42PD1-P24 vaccines or pVAX1 vector intramuscularly plus electroporation, then euthanized two weeks after the 2nd immunization (Fig. 1B). Anti-p24 antibody response in Δ42PD1-P24 immunized group was almost 100-fold higher than that of P24 group detected by ELISA for total IgG (Fig. 1C). For T cell responses, IFN-γ-producing cells were measured via ELISpot assay using stimulants of p24 peptide pool or peptides specific for CD4 and CD8 T cells. The Δ42PD1-P24 vaccine was highly immunogenic and elicited robust CD8 T cell responses compared to the P24 vaccine (5.3-fold), while no significant difference was observed for CD4 T cell responses between the two groups (Fig. 1D). Furthermore, we examined mice peripheral blood for vaccine induced CD8 T cell response using p24-specific H-2K^d^ AMQMLKETI-tetramer by flow cytometry. Consistently, the frequency of tetramer^+^ CD8 T cells in Δ42PD1-P24 immunized mice was significantly higher than that in P24 vaccine immunized mice (Fig. 1E, F). These data demonstrated that fusion expression of Δ42PD1 enormously increased DNA vaccine-elicited p24-specific immune responses.

Given the robust immune responses induced by the Δ42PD1 fusion DNA vaccine, we sought to evaluate its protective efficacy in BALB/c mice. Two weeks after the 2nd immunization, mice were challenged with AB1-Gag tumor cells, a murine mesothelioma cell line stably expressing HIV-1 Gag (Fig. 1B). Notably, the tumor growth rate was strikingly lower for Δ42PD1-P24 vaccine group compared with that for P24 and pVAX1 control groups (Fig. 1G). Mice were euthanized 3 weeks-post challenge and tumors were removed. Strikingly, only one tumor with ignorable size was acquired from the Δ42PD1-P24 vaccine group (Fig. 1H). Our data indicated that the Δ42PD1-P24 vaccine almost completely protects mice against tumor challenge, which is significantly more efficient than the P24 vaccine at eliminating implanted AB1-Gag cells.

To verify whether Δ42PD1 amplifies immune responses when provided separately instead of fused together, we constructed Δ42PD1-expressing plasmid by removing the p24 coding sequence from the Δ42PD1-P24 vaccine, and then vaccinated BALB/c mice with the mixture of P24 vaccine and Δ42PD1-expressing plasmid (Fig. 1B). The p24-specific immune responses were assessed by ELISA for antibody level and ELISpot for specific T cell counts. Compared with the P24 vaccine alone, the anti-p24 antibody level and the p24-specific T cell counts showed no significant increase in the Δ42PD1 mixed group (Fig. 1I, J). We, therefore, concluded that the fusion expression of Δ42PD1 with immunogen is essential for eliciting greatly strengthened humoral and cellular immune responses.

Given that Δ42PD1 interacts with dendritic cells via engaging TLR4 [10–12], we speculate that TLR4 plays an essential role in improved efficacy for Δ42PD1 fusion expression DNA vaccine. To test this hypothesis, TLR4 knockout mice generated by CRISPR/Cas9 technology were immunized with Δ42PD1-P24 vaccine, P24 vaccine and pVAX1 vector following the up-mentioned vaccination schedule (Fig. 1B). As expected, Δ42PD1-P24 vaccine lost its advantages in TLR4 knockout mice, eliciting comparable levels of p24-specific plasma antibodies and IFN-γ-producing T cells (Fig. 1K, L). These data demonstrated that the enhancement of the immunogenicity of the Δ42PD1-P24 DNA vaccine is dependent on TLR4 on DCs.

## Discussion

The first proof of the concept of a DNA vaccine was made in 1990 and involved the injection of DNA constructs expressing reporter genes into the mouse skeletal muscle [15]. The first DNA vaccine for veterinary use was approved as early as 2005 to protect horses from West Nile virus. Human applications of DNA vaccines have lagged behind, largely due to the sub-optimal immunogenicity when compared to traditional vaccine approaches. The present study clearly shows that a DNA vaccine could be configured to improve its immunogenicity.

To improve the immunogenicity of DNA vaccine, various strategies have been tested including vector design, antigen codon optimization, electroporation, use of traditional and molecular adjuvants, co-expression of molecular adjuvants and prime-boost regimen [9]. We made a construct in which the vaccine protein was fused to the extracellular domain of Δ42PD1 that would engage the TLR4 receptor on DCs following secretion from the transfected muscle [11, 12]. Here, we found that the Δ42PD-fusion DNA vaccine resulted in a large increase in both humoral immunity and cellular immunity in vivo and in the efficacy of protecting mice from challenges with tumor cells expressing the vaccine protein. Zhou et al. have previously reported the use of Δ42PD1 to improve the DNA vaccination [10]. However, the earlier study did not reveal the underlying mechanisms. We modified the earlier vaccine constructs here by removing the CH2-CH3 domain of rabbit IgG to eliminate the potential impact on vaccine immunogenicity, and by removing the Δ42PD1 inherent signal peptide and the linker between Δ42PD1 and HIV-1 Gag p24 to minimize the size of the constructs. These modifications did not compromise the expression of the vaccine protein. Importantly, after modification, the adaptive immune responses and protective efficacy were consistently enhanced by Δ42PD1-fusion expression, highlighting that Δ42PD1 alone is powerful enough to improve the immunogenicity of DNA vaccine when fused with vaccine antigen. In addition, our data demonstrated that Δ42PD1 dramatically augments DNA vaccine-elicited adaptive immunity in a TLR4-dependent manner in vivo.

TLR4 belongs to the pattern recognition receptor family, which plays a fundamental role in pathogen recognition, activation of the innate immunity and initiation of the adaptive immunity [16]. Early studies demonstrated that lipopolysaccharide (LPS), a TLR4 agonist derived from gram-negative bacteria, had a potent antibody-enhancing property when given in conjunction with protein antigens [17]. Further studies revealed that using TLR4 agonist in the vaccine formulation increases the diversity of the variable region sequences of the antigen-targeting antibodies and correlates with improved antigen neutralization [18]. Mechanistically, TLR4 signaling triggers inflammasome activation and then drives the production of IFN-γ by innate cells that, in turn, promotes the TH1 immunity [19]. Although Δ42PD1, a novel endogenous TLR4 agonist, triggers the secretion of pro-inflammatory cytokines [11], our data showed no improvement in adaptive immunity when given in combination with P24 DNA vaccine instead of fusion expression. One possible reason for this observation is the application of electroporation as a delivery method, which causes tissue damage and subsequently strong inflammatory responses [20, 21]. Speculatively, the extensive inflammatory response induced by electroporation could minimize the effect of inflammatory cytokines mediated by Δ42PD1-induced immune responses. Our results highlight the superiority of Δ42PD1 co-expressing DNA vaccine formulation and the crucial role of Δ42PD1-TLR4 interaction in improving the immunogenicity of Δ42PD1 fusion DNA vaccine. So, it is unsurprising that this improvement was not observed in TLR4 knockout mice.

The initiation of cytotoxic immune responses by DCs requires the presentation of exogenous antigenic peptides to CD8 T cells, a process called cross-presentation. TLR4 engagement induces re-organization of lysosomal distribution that delays antigen degradation to transiently enhance cross-presentation, thereby optimizing CD8 T cell responses [22]. DNA vaccines mainly transfect muscle cells resulting in poor antigen cross-presentation due to a lack of co-stimulation. Theoretically, when the vaccine protein was fused to Δ42PD1, it could target TLR4 on DCs following secretion from transfected muscle cells, thereby improving antigen cross-presentation and augmenting CD8 T cell responses. Consistently, our data show a marked increase in CD8 T cell responses elicited by Δ42PD1-fused DNA vaccine and improved protective efficacy in vivo. Interestingly, although the CD4 T cell response elicited by Δ42PD1-fused DNA vaccine was also slightly enhanced, it did not reach a statistically significant level. This observation could at least partially be explained by antigen cross-presentation. The enhanced cross-presentation would correspondently reduce antigen presentation to CD4 T cells, leading to a relatively weak CD4 T cell response. Collectively, we propose that the strategy of DC targeting via TLR4 or perhaps other receptors in the context of DNA vaccination will significantly enhance efficacy and may be particularly valuable for protection against chronic infectious diseases and emerging infectious diseases such as AIDS and COVID-19.

## Supplementary Information

**Additional file 1: Figure S1**. Verification of the antigen expression post-transfection of vaccine constructs in HEK-293T cells.

## Data Availability

All data generated or analyzed during this study are included in this published article. Original material is available from the corresponding author upon reasonable request.

## Abbreviations

HIV-1: Human immunodeficiency virus type 1
SARS-CoV-2: Severe acute respiratory syndrome coronavirus 2
COVID-19: Corona virus disease 2019
DC: Dendritic cell
PD-1: Programmed cell death protein 1
TLR4: Toll-like receptor 4
ORFs: Open reading frames
IFN-γ: Interferon gamma
