# Supplementary material for: A Δ42PD1 fusion-expressing DNA vaccine elicits enhanced adaptive immune response to HIV-1: the key role of TLR4
Source: Virol J. 2022 Nov 1;19:174. doi: 10.1186/s12985-022-01909-9 (PMC9628179; doi:10.1186/s12985-022-01909-9)
Supplement: Supplementary file 1 — Additional file 1: Figure S1. Verification of the antigen expression post-transfection of vaccine constructs in HEK-293T cells. [file 12985_2022_1909_MOESM1_ESM.docx]

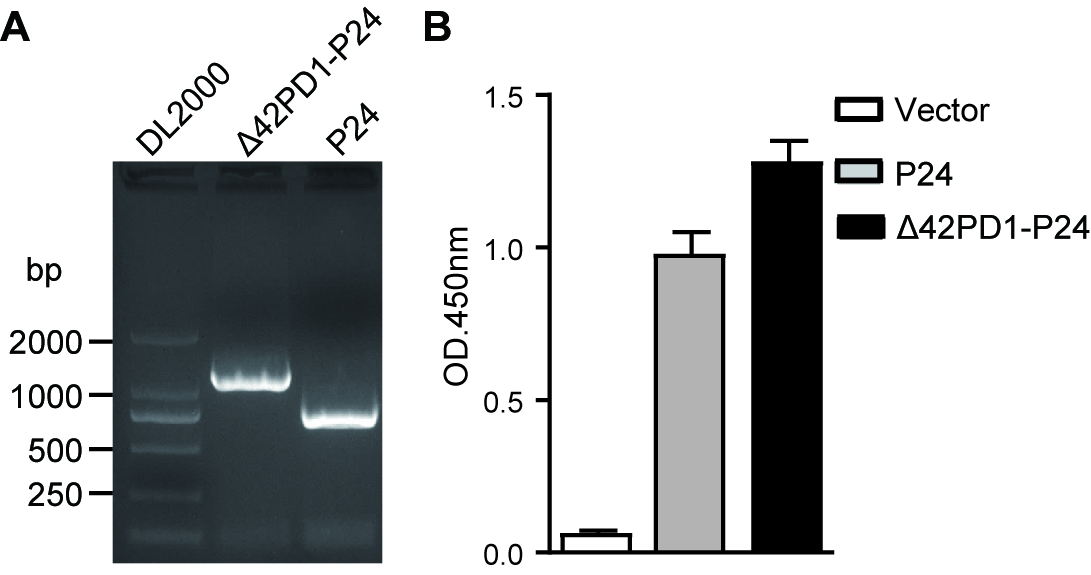


**Figure S1. The expression of the p24 antigen in HEK-293T cells.**

(A) The transcription of p24 and Δ42PD1-p24 in HEK-293T cells was determined via RT-PCR two days post-transfection of indicated vaccine plasmids. (B) The p24 antigen in HEK-293T culture media was detected via ELISA two days post-transfection of indicated vaccines or pVAX1 (Vector). Data were presented as mean ± SEM from three independent experiments.
